# Exploring the Ramifications of Delayed Hospital Discharges: Impacts on Patients, Physicians, and Healthcare Systems

**DOI:** 10.7759/cureus.61249

**Published:** 2024-05-28

**Authors:** Kanishk Aggarwal, Bhupinder Singh, Himanshi Banker, Mason T Stoltzfus, Jinpyo Hong, FNU Anamika, FNU Nishkamni, Jaskaran Munjal, Rohit Jain

**Affiliations:** 1 Internal Medicine, Dayanand Medical College and Hospital, Ludhiana, IND; 2 Internal Medicine, Government Medical College, Amritsar, IND; 3 Medicine and Surgery, Maulana Azad Medical College, Delhi, IND; 4 Neurosurgery, Penn State University College of Medicine, Milton S. Hershey Medical Center, Hershey, USA; 5 Internal Medicine, University College of Medical Sciences, Delhi, IND; 6 Internal Medicine, Government Medical College, Jammu, IND; 7 Internal Medicine, Shri Ram Murti Smarak Institute of Medical Sciences, Bareilly, IND; 8 Internal Medicine, Penn State Health Milton S. Hershey Medical Center, Hershey, USA

**Keywords:** hospital-acquired infections, bed blocking, hospitalization, early discharge, delayed discharge

## Abstract

Prolonged hospital stays can significantly impede patients' recovery, negatively affecting anything from physical health via issues like hospital-acquired infections and increased complications due to immobility to psychological health. Several studies investigated the psychosocial impact of prolonged hospital stays, revealing a variety of patient perspectives, such as feeling uncertain and frustrated about their conditions, which can erode their trust in healthcare providers. Delayed discharges not only affect patients but also have multifaceted effects on healthcare providers, potentially reducing physician efficiency and contributing to higher rates of burnout among healthcare professionals. This article investigates the consequences of delayed versus early discharge on physicians, patients, and the overall hospital system. We conducted an extensive search through PubMed and Google Scholar using the keywords "delayed discharge," "hospital discharge," and "bed blocking" to identify all the recent studies highlighting the dynamics of patient discharge. Our results support the hypothesis that reducing delayed discharge rates will not only improve patient outcomes but also have widespread fiscal impacts. This review also outlines measures to reduce delayed discharges, ultimately leading to a significant enhancement in the healthcare system.

## Introduction and background

Advancements in healthcare technology have been associated with early disease detection and increased healthcare access, resulting in a higher hospitalization rate. In 2019, the expenses associated with hospitalization were significant, with an average adjusted cost of $14,101 for each patient's community hospital stay. The most common reasons for hospitalization include septicemia, heart failure, pneumonia, and diabetes mellitus [1]. The complexity of healthcare options, including treatments, diagnostics, and care management, increases the difficulty of providers’ medical decision-making. One consequence of this complexity is the issue of "delayed discharge," where patients remain in the hospital due to their healthcare teams' failure to optimize care coordination across multiple disciplines [2]. Delayed discharge, also known as "bed blockage," is the inappropriate occupation of hospital beds by patients who could have been discharged earlier with optimized care coordination and plans. This seriously affects hospitals' capacity to accommodate waiting patients and provide efficient treatment [3]. According to a study, nearly 30% of older individuals experience delays in their hospital discharge, exposing them to additional hospital-related risks, creating emotional and physical dependency, incurring additional hospital costs, and limiting the availability of inpatient beds [4]. The factors contributing to these delays include insufficient communication between healthcare and social care, inadequate assessment and preparation for discharge, insufficient advance notice of discharge, limited engagement of patients and their families, over-reliance on informal caregiving, and the neglect of the specific needs of vulnerable groups [5]. Delays in patient discharge have a knock-on impact, limiting the availability of hospital beds, causing delays in ambulance handovers, increasing the financial burden for patients, increasing the workload of healthcare professionals, and slowing the admission of patients from emergency departments (EDs) [6]. Table 1 summarizes the advantages and disadvantages of early discharge versus delayed discharge.

**Table 1:** Early discharge vs. delayed discharge

Aspects	Early discharge	Delayed discharge
Advantages	Reduces healthcare cost	May reduce chances of rehospitalization
	Reduced risk of hospital-acquired infections	May strengthen the physician-patient relationship
	Increases hospital bed availability	May increase patient safety
	Reduces physician's workload	
Disadvantages	May lead to premature discharge and inadequate recovery	Increases physician’s workload
	May increase the risk of hospitalization	Increases healthcare cost
		Blocks bed for incoming patients
		Hospital overcrowding

The limited existing research on delayed discharges in healthcare reveals a range of downstream effects on multiple stakeholders, including physicians, medical personnel, hospitals, and caregivers. A study examined these impacts, uncovering how some staff became overly focused on discharges, diverting attention from current patient care. Staff attitudes toward discharges varied due to external pressures from managers and senior physicians to expedite the process. Delays also harmed working relationships and information sharing among healthcare professionals. In summary, delayed discharges significantly affected interprofessional relationships [7].

A separate study, which examined the perspectives of patients and caregivers affected by delayed discharge, found that its effects primarily resulted in psychological uncertainty, resulting in a decreased level of trust in the healthcare system. This study delved into how delayed discharges could contribute to a pessimistic outlook on the patient's future, frustration due to extended wait times for placement in long-term care (LTC) facilities and nursing homes, and a lack of clarity about the reasons for their prolonged hospital stay [8]. These psychological, social, and physical impacts on patients' well-being could create a detrimental cycle, ultimately reducing the rates of timely discharges. As a result, the affected healthcare system could suffer from hospital bed shortages and higher workloads, jeopardizing its effectiveness and efficiency.

While some of the aforementioned studies have investigated delayed discharges, there is still a considerable gap in the research addressing how to successfully manage this issue, particularly regarding insurance and ethical considerations. Among the major concerns are rising insurance costs and potential infringements on patient autonomy. The absence of standardized guidelines hinders efforts to prevent delayed discharges. Initiatives should be established and seamlessly integrated across different sectors, including hospitals, primary care, and home and community care. This integrated approach is essential to address the root causes of delayed discharges and prevent situations where resolution in one sector merely shifts the issue to another [9]. It is critical to emphasize that while attempting to lessen the burden of delayed discharges, the quality of patient care should not be compromised, and clinical judgment must remain robust. Distinguishing between patients who would benefit from an extended hospital stay and those who may be safely discharged is crucial. This article aims to shed light on the complexities surrounding delayed discharges, examine their core causes, identify systemic weaknesses, and devise strategies to lessen the occurrence of delayed discharges. The ultimate goal is to have a positive impact on healthcare systems’ fiscal and patient care outcomes.

## Review

Delayed discharge

Impact of Delayed Discharge on Patient

Delays in discharge from a hospital or rehabilitation setting could have a significant impact on the health of patients who have been admitted for the management of their illnesses. The majority of the consequences of delayed discharge are found to have unfavorable effects, which could include increased risk of hospital-acquired infections, psychological stress, loss of independence, decreased social support, and ultimately reduced overall quality of life [7,10]. Delayed discharge is strongly associated with an increased incidence of nosocomial infections, which may include infections involving multidrug-resistant organisms like methicillin-resistant *Staphylococcus aureus* (MRSA), *Clostridium* *difficile*, and *Escherichia coli* [11,12]. According to Jeon et al., a one-day increase in the duration of stay positively correlated with increased odds of developing bloodstream infections with an odds ratio of 1.025 (95% confidence interval [CI], 1.023-1.027) [12]. A study by Macedo-Viñas et al. highlighted that nosocomial infections should be treated on time and have an estimated 1.26-fold higher average cost of treatment for the patients infected with MRSA [13]. It is also important to consider that infections due to delay in discharge can further extend the length of stay (LOS), particularly in elderly and severely ill patients. Most of the studies reviewed examining the relationship between LOS and nosocomial infections found a direct relationship.

Delayed discharges can have financial implications for patients and their families, including extra co-pays, deductibles, and coinsurance for additional or extended services like nursing care, medications, and room fees [14]. A study conducted in Massachusetts identifying causes of delayed discharge of trauma patients reported that the mean hospitalization cost for 32 patients admitted for prolonged stays (excessively prolonged hospitalization [ExProH]) was $54,646, greater than the mean hospitalization cost for non-ExProH patients ($18,444). In just 20% of the cases, clinical grounds were given for the delays in discharge. The remaining discharges were unduly delayed due to difficulty finding appropriate rehabilitation centers (47%), delays in hospital operations (26%), or problems with payers (7%) [15]. Independent of the cause of delayed discharge, an extra bed day can have serious repercussions, including a 30.7% increase in daily expenses [7].

A more holistic approach to evaluating the effect of delayed discharge from healthcare settings can be achieved through the patient satisfaction scoring system. Patient satisfaction scores affect hospital reimbursement and status. A retrospective review by Diwan et al. suggested that delayed discharge is linked to higher odds of low patient satisfaction and decreased odds of recommending the hospital [16]. However, many other studies have found no correlation between patient satisfaction scores and LOS. Borghans et al., on the other hand, found no such correlation in six out of seven specialties, including internal medicine, cardiology, pulmonology, neurology, general surgery, orthopedic surgery, and obstetrics and gynecology, over the period of seven years. Pulmonology was the only specialty in which patient satisfaction inversely correlated with the LOS [17]. This stratification provided a better overview of how to carefully consider the inpatient dynamics affecting patient satisfaction instead of LOS alone. In the pulmonology cohort, patients with chronic lung diseases were managed for symptom relief and disease progression prevention. They were not fully involved in decision-making due to the complexity of the disease and the involvement of multiple specialists, indirectly delaying the discharge. Patients in the other specialties were well-informed and had better satisfaction, irrespective of the LOS [17]. Therefore, with higher quality of care and patient participation, the patients exhibited greater overall satisfaction. Stronger patient-physician relationships also correlate with higher patient satisfaction despite longer LOS. Longer LOS may provide time for increased relationship-building between healthcare professionals and patients, improving patient satisfaction [18]. It is worth noting that satisfaction scores related to patient discharge are subjective and influenced by many factors.

Impact of Delayed Discharge on Hospital

In addition to increased risk for adverse outcomes, including accelerated functional decline, delirium, pressure ulcers, nosocomial infections, and falls with unnecessary and prolonged hospital stays, delayed discharges often contribute to additional patient and family stress, stigmatization, and patient safety challenges due to hospital overcrowding and reduced accessibility to acute care resources. Furthermore, delayed discharges increase hospital and health system costs through the expenditure of resources that could be provided more efficiently in alternative settings such as home care or LTC [19].

EDs are intended for quick triage, stabilization, and initial treatment of patients. However, when admitted, patients no longer require the expertise of ED personnel but are waiting for transfer to the appropriate ward for continued care, which results in ED boarding or "gridlock" in the ED [20,21]. EDs then become sites for ongoing (i.e., longitudinal) care in the acute phase of hospitalization, often leading to delayed discharges. A study showed that patients who are admitted to the hospital and wait in the emergency department for more than a day before being admitted tend to have extended hospital stays and incur 10% to 20% higher costs than patients admitted promptly [20]. Additionally, a study conducted by Forster et al. concluded that the LOS in the ED is significantly affected by hospital occupancy. This study revealed that a 10% increase in hospital occupancy led to an 18-minute increase in the LOS in the ED [22]. Bagust et al. drew attention to the fact that acute hospitals operating at 90% or higher bed occupancy levels are more likely to experience regular bed crises, which poses a risk to patients [23]. Another study by Mustafa et al. demonstrated a statistical association between ED boarding and delayed discharge [20]. Delayed discharge due to ED boarding often increases the hospital's financial burden. According to Krochmal and Riley, patients with a longer ED LOS tend to have extended hospital stays, leading to increased hospital costs. They estimate that an extended ED LOS can result in an additional $6.8 million in hospital costs over three years [24]. Another study by Foley et al. showed that if ED boarding was eliminated from the hospital, the county hospital could have saved $9.8 million in charges, and the university hospital could have saved $3.85 million in costs over a year. It also showed that payers are less likely to cover fees for extended inpatient stays, which means that a substantial portion of these charges and costs may go unpaid, increasing the hospital's net cost [25]. A study by Bayley et al. examined the additional cost of extended ED LOS for chest pain patients waiting for non-intensive care unit (ICU) monitored beds and found that 91% of admitted patients waited over three hours for an inpatient bed, translating to a potential revenue loss of $204 per patient [26]. Similarly, Falvo et al. found a significant loss of potential hospital revenue and ED functional treatment capacity associated with increased length of ED stays of admitted patients. The study concluded that transferring admitted patients from the ED to an inpatient unit within 120 minutes would have increased the functional treatment capacity of the ED by 10,397 hours over one year. Furthermore, by reducing admission process delays during their 12-month study, the hospital could have accommodated another 3,175 patient encounters in its existing treatment spaces. The authors concluded that the hospital could have generated $3,960,264 in net revenue by providing emergency services to new patients in ED beds used to board admitted patients for one year [25,27].

In addition to ED boarding, a lack of rehabilitation beds accounts for delayed hospital discharge and additional costs, as demonstrated by Hendy et al.’s study involving 83 consecutive patients who were admitted for 888 days. The study found that 65% of patients experienced delays while waiting for a rehabilitation service, and 48% had delays extending their discharge date. These delays accounted for 21% of the inpatient stay, which cost approximately £565 per patient. Furthermore, 77% of these holdups were due to delays in providing social and therapy requirements. Delayed discharges can be costly for hospitals and can be a depressing experience for patients [19,28]. While most economic evaluation studies have focused on trauma, surgical, and ICU patients, the findings are consistent with the increased costs associated with delayed discharges. In addition to the higher fiscal cost, the shortage of beds also increases the Sequential Organ Failure Assessment score while waiting, reflecting a worsening of organ dysfunction during this period, as suggested by the study by Cardoso et al. [29]. It has been reported that in countries such as the United States, a delay of more than six hours in transferring patients to the ICU can lead to an increase in hospital LOS and ICU patient mortality. According to a study by Young et al., patients with four or more hours of delay to treatment after physiological deterioration were found to have a 3.5 times higher nonadjusted mortality rate. However, there are conflicting data in this respect, as a study by Cardoso et al. found no correlation between delayed admission due to the nonavailability of beds and LOS but found increased mortality related to delayed admission [29-31]. A study by Teklie et al. found that almost half of critically ill patients (54.9%) experienced delays in being admitted to the ICU due to the unavailability of ICU beds. Other reasons for the delays included late results of radiological investigations (15.1%), poor prognosis (9.3%), delayed laboratory investigation results (7.0%), and delayed therapeutic procedures that can be performed in the ED (3.5%). This study is consistent with a previous study conducted in Ethiopia, which identified a shortage of ICU beds as the primary reason for the prolonged stay of patients in the ED [32]. Additionally, there may be conflicts of interest between healthcare professionals and procedural delays due to waiting for tests and investigations. In some cases, confusion and redundancy in the plan of care can also contribute to delays [7].

Insurance coverage can also lead to delayed discharges as a disproportionate number of Medicaid and Medicare patients experience delayed discharge. For instance, in California, Medicaid has one of the lowest reimbursement rates in the United States, which makes it less attractive for postacute care facilities such as skilled nursing facilities to accept patients on Medicaid. A study by Cai et al. found that patients with private insurance were less likely to experience discharge delays, indicating higher reimbursement rates. Moreover, most of these delays were related to placement issues, suggesting rehabilitation centers may select patients based on their insurance status [33].

Impact of Delayed Discharge on Physician

Bed blocking strains healthcare systems and could impact the performance of healthcare providers, particularly physicians. Due to delayed discharges, physicians must care for more patients, increasing stress and workload. In a study by Watson et al., delayed discharges were found to be one of the factors increasing physician workload and contributing to burnout. Delayed discharges increased the odds of physicians experiencing emotional exhaustion by 6.37 (95% CI: 2.94-13.82; p < 0.0001) [34]. However, it is unclear if the providers' suboptimal care leads to bed blocking. There is not enough research conducted on how physicians’ performance is affected by delayed discharges and how this may, in turn, affect the physician-patient relationship. The impact of delayed discharge on physicians is underrated in the current healthcare setting, and future research in this area may uncover hidden factors affecting physicians' health and functioning.

Early discharge

Timely discharge from a hospital setting has shown promising benefits in several studies with positive effects on patient health and the ergonomics of the healthcare setting. However, early discharge should be based on strict medical assessment by physicians and should not compromise the patient's healthcare outcomes. A team of physicians, nurses, and therapists can assist with and organize the early discharge process, and patients may leave the hospital sooner than usual and undergo more rehabilitation in the comfortable setting of their own homes [35]. In a review by Fearon and Langhorne, 14 trials with 1,957 participants were identified, finding that patients who received assistance with early discharge went home quicker and were more likely to stay at home and restore independence in everyday activities [36]. Another modality that can aid the early discharge process of patients is the concept of hospital at home (HaH). An early discharge to HaH offers patients active treatment by medical professionals in their homes for conditions that would normally necessitate inpatient care [37]. In a study by Kanagala et al., individuals receiving care for HaH had a decreased readmission rate and median LOS. Patients in HaH experienced better clinical results than those in a hospital setting. According to this study, the HaH unit offers a scalable, adaptable, and integrated platform that can be inexpensively adjusted to high-demand scenarios [38]. The patient's discharge to HaH can later be monitored for daily evaluation either in-person or via telehealth services, and hence, their progress can be regularly tracked [38]. However, care should be taken in older patients with multiple comorbidities because early discharge at home may increase the risk of readmissions for older people with a mix of conditions [37].

A study evaluating the postnatal discharge of mothers and infants found possible benefits of releasing women and their infants from the hospital earlier, such as better sleep and familiar surroundings, less exposure to the artificial routines in the hospital, and a lower risk of infection. However, while assessing readmission rates of mothers and infants individually after early discharge, the study found a slightly higher chance of infant readmission in 28 days with conditions like jaundice, dehydration, and infections with a risk ratio (RR) of 1.59 (95% CI: 1.27-1.98) [39]. The readmission rate was slightly increased, but the 28-day risk of mortality in early discharged infants was unclear due to an insignificant RR of 0.39 (95% CI: 0.04-3.74) [39]. From a financial perspective, early discharge was found to slightly decrease the cost of hospital care from the time of childbirth to postnatal discharge. A study by Kumar et al. evaluating early discharge of mild acute pancreatitis patients to outpatient facilities demonstrated that by discharging patients with mild acute pancreatitis on day 2 through a well-organized clinical strategy, they could reduce the financial costs and improve patient satisfaction. The authors formulated a statistically significant evidence-based six-factor protocol, which enabled them to predict the LOS for more than two days. Patients with a score suggesting an LOS requiring no more than two days were discharged by the end of the second day. The implication of a protocol that could predict the LOS significantly led to the reduction of bed-day and healthcare finances. The estimated cost of inpatient care per patient per day of $1222 was significantly cut down to $779 by early discharge to an outpatient facility [40].

Future implications

Managing delayed discharge from hospitals is challenging and requires various measures to address it. While developing and implementing initiatives to improve delayed discharges, it is crucial to explore the needs and experiences of patients, their families, and providers. As per the recommendations of healthcare providers and authors such as Cressman et al., involving patients and their families in developing and writing best practice guidelines is crucial. It is also essential to provide patients and their families with accurate and timely information, include them in all decision-making processes, and create a forum to address their questions and concerns regarding the discharge process and estimated discharge date [41,42]. To include such concrete, actionable steps, Cadel et al. developed a framework with 12 leading practices to reduce these discharge delays. Most initiatives resulted in positive outcomes, such as decreased hospital stay duration and LOS [9].

In addition, other initiatives that can be employed to target delayed discharge, like more focus on common practice change initiatives, include hospital-based, nurse-led discharges and cross-sectoral transitional programs such as home first, discharge to assess, and hospital to home [9]. Graham et al., in their retrospective study comparing nurse-led and doctor-led discharges (standard discharge pathway) of postlaparoscopic surgery patients, showed that patients in the nurse-led group were significantly more likely to be discharged on the day of surgery. The study found that the success of the nurse-led model was not due to patient factors but rather the quick availability of the nurse specialist who could implement the discharge criteria (specifically for nurse-led discharge) much faster than the doctor-led group who did not use such criteria [43]. In another observational study conducted by Blecker et al., it was found that increasing weekend staff, such as hospitalists, care managers, and social workers, resulted in a decreased average LOS and an increased proportion of weekend discharges [44]. This highlights the need for a multifaceted and collaborative approach involving multiple stakeholders and team members to create avenues and train staff during patient handover and focus on patient flow throughout the hospital to address the numerous factors that impact delays [8].

Delayed discharges from hospitals can also be reduced using statistical and predictive modeling techniques. These models can explore the impact of various initiatives, such as increasing the supply of nursing home beds, developing potential care pathways for geriatric patients, and analyzing reimbursement costs associated with discharge strategies [9]. According to empirical analysis and modeling by Gaughan et al., increasing the supply of LTC beds can help decrease delayed discharges resulting from a lack of social care [45]. Another study by McCloskey et al. and Wilson et al. recommends strategies such as facilitating the subsidization of home care, increasing the number of LTC beds, and developing an LTC wait-list plan. Additionally, policies should be implemented to involve patients awaiting placement in making decisions about their placement. Finally, advocacy for home care services for patients awaiting placement should be increased to address delayed discharge concerns [8,46,47].

## Conclusions

Delayed discharge, also referred to as "bed blockage," involves the inappropriate utilization of hospital beds leading to substantial adverse effects on a hospital's capacity to reduce waiting lists and provide efficient treatment. Contributing factors include inadequate communication between healthcare and social care, insufficient discharge assessment and preparation, insufficient notice of impending discharge, limited involvement of patients and their families, excessive reliance on informal caregivers, and the neglect of the unique requirements of vulnerable groups. Limited research on delayed discharges in healthcare has unveiled diverse impacts on various stakeholders, including physicians, medical staff, hospitals, and caregivers. Prompt discharge from a hospital has shown positive outcomes in multiple studies, benefiting patient health and healthcare facility operations. Nevertheless, early discharge should be grounded in rigorous medical assessment to ensure patient health is not compromised. Therefore, the complexity of this issue underscores the necessity for further research to gain a deeper understanding of the true effects of early and late discharges on patients, physicians, and healthcare systems.
